# Influence of vector design and host cell on the mechanism of recombination and emergence of mutant subpopulations of replicating retroviral vectors

**DOI:** 10.1186/1471-2199-10-8

**Published:** 2009-02-09

**Authors:** Matthias Paar, Dieter Klein, Brian Salmons, Walter H Günzburg, Matthias Renner, Daniel Portsmouth

**Affiliations:** 1 Institute of Virology, Department of Pathobiology, University of Veterinary Medicine, Vienna, Austria; 2 Christian-Doppler Laboratory for Gene Therapeutic Vector Development, Vienna, Austria; 3 Vetomics – Core Facility for Research, University of Veterinary Medicine, Vienna, Austria; 4 Austrianova Singapore Pte Ltd, 20 Biopolis Way, #05-518 Centros Singapore 138668, Singapore

## Abstract

**Background:**

The recent advent of *murine leukaemia virus *(MLV)-based replication-competent retroviral (RCR) vector technology has provided exciting new tools for gene delivery, albeit the advances in vector efficiency which have been realized are also accompanied by a set of fresh challenges. The expression of additional transgene sequences, for example, increases the length of the viral genome, which can lead to reductions in replication efficiency and in turn to vector genome instability. This necessitates efforts to analyse the rate and mechanism of recombinant emergence during the replication of such vectors to provide data which should contribute to improvements in RCR vector design.

**Results:**

In this study, we have performed detailed molecular analyses on packaged vector genomes and proviral DNA following propagation of MLV-based RCR vectors both in cell culture and in pre-formed subcutaneous tumours *in vivo*. The effects of strain of MLV, transgene position and host cell type on the rate of emergence of vector recombinants were quantitatively analysed by applying real-time PCR and real-time RT-PCR assays. Individual mutants were further characterized by PCR, and nucleotide sequence and structural motifs associated with these mutants were determined by sequencing. Our data indicate that virus strain, vector design and host cell influence the rate of emergence of predominating vector mutants, but not the underlying recombination mechanisms *in vitro*. In contrast, however, differences in the RNA secondary structural motifs associated with sequenced mutants emerging in cell culture and in solid tumours *in vivo *were observed.

**Conclusion:**

Our data provide further evidence that MLV-based RCR vectors based on the Moloney strain of MLV and containing the transgene cassette in the 3' UTR region are superior to those based on Akv-MLV and/or containing the transgene cassette in the U3 region of the LTR. The observed discrepancies between the data obtained in solid tumours *in vivo *and our own and previously published data from infected cells *in vitro *demonstrates the importance of evaluating vectors designed for use in cancer gene therapy *in vivo *as well as *in vitro*.

## Background

Retroviruses undergo a high frequency of recombination events during replication of their genomes, leading to a large number of variants or "quasispecies" in a viral population (reviewed in [1-5]). The majority of recombinations arise during reverse transcription of the retroviral RNA genome into proviral DNA by the retroviral reverse transcriptase (RT), primarily during minus-strand DNA synthesis, although the cellular RNA polymerase (RNAP) II, which synthesizes the retroviral genome, has also been demonstrated to introduce mutations into retroviral vector genomes at a very low frequency [6,7]. Not only does the retroviral RT lack proof-reading activity, which leads to a high rate of base misincorporations (reviewed in [8] and [4]), but it also exhibits low processivity and frequently dissociates from its RNA template, which can in turn lead to template-switching both within the same molecule (intramolecular recombination) [9-11] and between the two co-packaged molecules of the viral RNA dimer (intermolecular recombination) [12,12,15].

Retroviruses have been used for a wide variety of gene-delivery applications in both pre-clinical and clinical studies, including the treatment of solid tumours [16,17]. Until recently, cancer gene therapy applications of retroviruses were restricted to the use of replication-deficient retroviral (RDR) vectors, but, since it has emerged that clinically beneficial transduction efficiencies are difficult to achieve with non-replicating vectors [18,19], attention has switched to the use of their replication-competent counterparts. Replication-competent retroviral (RCR) vectors based on *murine leukaemia virus *(MLV) have subsequently been shown to be powerful tools for gene transfer both in cell culture [20-26] and in solid tumours in pre-clinical studies [23,27-32].

A potential drawback with the use of RCR vectors, however, is that the expression of heterologous genes necessitates the elongation of the viral genome beyond its natural size, which can lead to a reduction in replication efficiency [33]. Moreover, due to the propensity of retroviruses to undergo recombination, deletion of heterologous sequences may occur, leading to the generation of mutants with increased replication efficiency relative to the parental vector, and, if this difference is great enough, the mutants will soon become dominant in a population of replicating viruses. This is doubly problematic for the success of gene therapeutic applications, since, not only do these mutants no longer express the therapeutic gene, but, due to superinfection resistance [34], cells infected with recombinant vectors become refractory to subsequent infection with the parental vector. It is hence of paramount importance to analyse the selective pressure for transgene deletion exerted on vectors of different design and under different propagation conditions, to allow a clear assessment of both the factors leading to the emergence of predominating vector mutants and the recombination mechanisms associated with their generation.

The underlying molecular mechanisms resulting in individual mutational and recombinatorial events have been extensively investigated in replication-deficient retroviral (RDR) vectors, but relatively few studies have investigated RCR vectors in this respect. In contrast to studies with RDR vectors, where recombination events taking place in a single replication cycle are detected, during multiple replication cycles of RCR vectors mutations can accumulate and individual mutations and deletions can be shuffled among the vector population by interstrand recombination. However, only recombinants which provide a replication advantage over the parental vector will become dominant in a population of replicating viruses, and hence analyses of sequences flanking recombination junctions of such vector mutants may provide information useful for RCR vector design.

In a recent study, we reported that the efficacy of MLV-based RCR vectors can be influenced not only by virus strain and transgene position, but, interestingly, also by the host cell [35]. In the present study, to shed more light on the possible mechanisms behind these differences, we have performed detailed molecular analyses on recombinants which emerged during vector replication both in cell culture and in solid tumours *in vivo*. The rate of emergence of vector mutants containing deletions in transgene sequences was quantified, and the underlying nucleotide sequence and structural motifs associated with the generation of such mutants were characterised.

## Results

### Propagation of MLV-based RCR vectors in vitro and in vivo

Very recently, data have been published unravelling the effects of virus strain, transgene position and target cells on the efficacy of Moloney (Mo)- or Akv-MLV-based RCR vectors genetically pseudoptyped with the 4070A amphotropic *env *gene, from which eGFP expression is mediated by the EMCV IRES [35]. The results revealed that vectors based on Mo-MLV and containing the IRES-eGFP cassette precisely fused directly to the 3' end of the MLV 4070A env gene replicate more efficiently and propagate a functional eGFP-expression over a longer time period than analogous vectors based on Akv-MLV, in which an identical IRES-eGFP cassette is also precisely fused to the 3' end of the MLV 4070A env gene, as well as Mo-MLV-based vectors containing the same IRES-eGFP cassette in the U3 region of the LTR. Furthermore, both Mo- and Akv-MLV-based vectors propagated eGFP expression for longer in HEK293 cells than in NIH-3T3 or U87-MG cells, regardless of transgene position. The loss of eGFP expression was presumably mediated by functional abrogation of the transgene cassette via recombination and/or point mutations gathered during vector replication.

**Figure 1:**
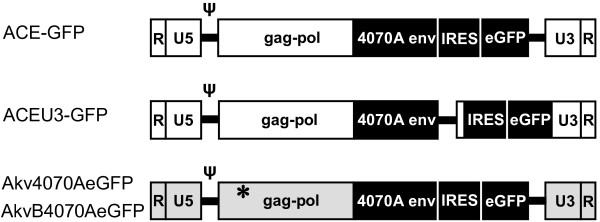
**MLV-based RCR vectors**. Moloney-MLV-based (ACE-GFP, ACEU3-GFP, white vector backbone) and Akv MLV-based (Akv4070AeGFP, AkvB4070AeGFP, grey vector backbone) vectors harbouring an IRES-eGFP transgene cassette either immediately downstream of the MLV 4070A *env *gene or inserted in the U3 region of the 3' LTR. A single amino acid was altered (Glu to Arg indicated by an asterisk) in the N-tropic vector Akv4070AeGFP to generate the B-tropic vector AkvB4070AeGFP, which can efficiently infect human cells.

Here, in the present study, we performed detailed molecular analyses to quantify the rate of the emergence of vector recombinants in populations of replicating MLV-based vectors, and to characterise the underlying recombination mechanisms leading to the generation of these mutants. The experimental design enabled each vector to maintain exponential spread throughout the duration of the experiment, as described previously [35]. Cells were inoculated with vectors ACE-GFP [22], ACEU3-GFP, Akv4070AeGFP or AkvB4070AeGFP [35] (Fig. 1) at an MOI of 0.001, and subsequently passaged every 2 or 3 days. Four days post infection, virus-containing supernatant was harvested and used to inoculate fresh cells, using a dilution factor resulting in infection levels of between 1% and 10%. This process was repeated for multiple serial infection cycles. The emergence of vector recombinants in solid tumours was investigated by infecting preformed subcutaneous U87-MG xenografts growing in nude mice.

### Detection and quantification of the emergence of vector recombinants in vitro and in vivo

**Figure 2:**
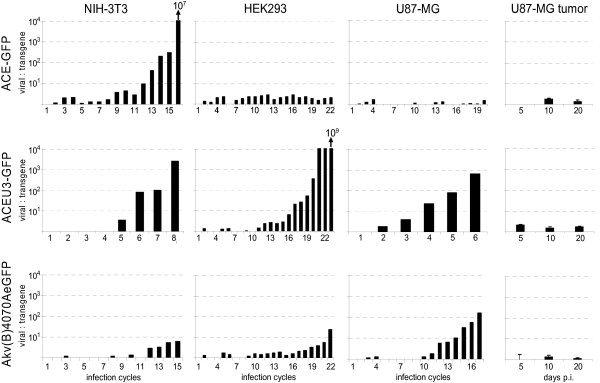
**Quantification of the rate of recombinant emergence by real-time PCR and real-time RT-PCR**. Viral RNA isolated from cell-free medium supernatant of *in vitro *infected NIH-3T3, HEK293, and U87-MG cells and DNA isolated from *in vivo *infected subcutaneous U87-MG tumours growing in Hsd:athymic Nude-*Foxn1*^*nu *^mice was used to determine the ratio of viral- and transgene-specific sequences in the packaged vector genome and in integrated proviruses, respectively. Two sets of primers and probes were used for real time PCR and real time RT-PCR, one annealing to sequences in the *env *gene and the second to sequences in the IRES or the *eGFP *gene, respectively. Viral to transgene specific sequence ratios are shown at the end of each infection cycle for cell lines infected *in vitro *and 5, 10, and 20 days post infection of solid tumours *in vivo*, respectively. Error bars represent the standard deviation of the viral to transgene ratios measured in infected solid tumours excised from 5 mice sacrificed at each time point.

The rate of emergence of vector recombinants lacking transgene sequences which were packaged into viral particles was quantitatively determined via real-time RT-PCR performed on RNA extracted from cell-free supernatants of infected cells at the end of each infection cycle. The emergence of vector mutants *in vivo *was quantified by real-time PCR performed on DNA extracted from infected tumours 5, 10, and 20 days following vector inoculation. Primers and probes annealing either to a region in the *env *gene or to a region in the IRES-eGFP transgene cassette were used, allowing discrimination between vectors which contain both *env *and transgene sequences, and those which contain the *env *coding region but no longer contain the complete transgene sequence. In the case of vector ACE-GFP replicating in NIH-3T3 cells, the ratio of *env *to transgene signal begins to increase at infection cycle 9, but does not increase at all in HEK293 or U87-MG cells infected with ACE-GFP (Fig. 2), indicating that no major deletion mutants emerge in these cells. The ratio of *env *to transgene signal in cells infected with vector ACEU3-GFP, on the other hand, increases in each cell line analysed, from infection cycles 5, 16, and 3 onwards in NIH-3T3, HEK293 and U87-MG cells, respectively (Fig. 2). Similarly, the ratio of *env *to transgene signal in cells infected with vector Akv4070AeGFP or AkvB4070AeGFP also increases in each cell line, albeit slightly later, from infection cycles 12, 19, and 12 onwards, in NIH-3T3, HEK293, and U87-MG cells, respectively (Fig. 2). By analysing the differences in the rate of increase of the ratio of *env *to transgene signal, it is apparent that vector mutants emerge most rapidly in cells infected with vector ACEU3-GFP, and that this process occurs most slowly in cells infected with vector ACE-GFP, except in NIH-3T3 cells. The rate of emergence of vector mutants was slowest, regardless of vector design or virus strain, when vectors were propagated in HEK293 cells. Significant increase in the ratio of *env *to transgene signal could not be detected in *in vivo *infected tumours, indicating that the majority of all vectors in the replicating virus population still contained the transgene cassette, even following 20 days of *in vivo *propagation (Fig. 2).

**Figure 3:**
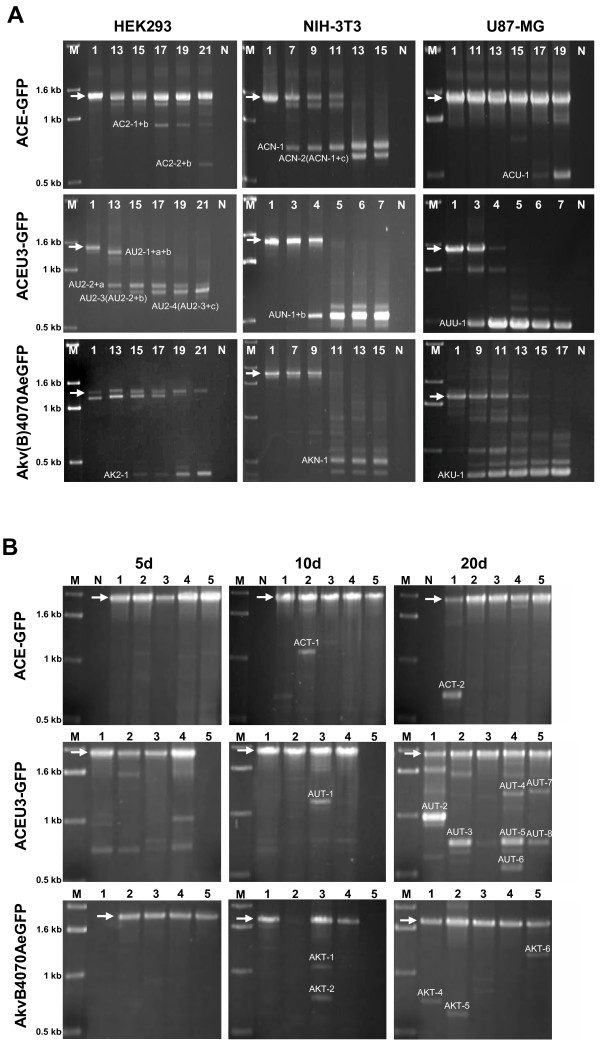
**Detection of deletion mutants by PCR**. Primers annealing to MLV 4070A *env *and LTR U3 specific sequences were used to amplify integrated proviral vector DNA isolated from **(A) ***in vitro *infected NIH-3T3, HEK293, and U87-MG cells and **(B) ***in vivo *infected subcutaneous U87-MG tumours growing in Hsd:athymic Nude-*Foxn1*^*nu *^mice. DNA was isolated from **(A) **different infection cycles or **(B) **from infected mice, indicated with numbers at the top of each lane, respectively. Nomenclature of PCR fragments is derived from the vector name as follows: the two-letter prefixes AC-, AU-, and AK- denote mutants derived from vectors ACE-GFP, ACEU3-GFP, and Akv4070AeGFP or AkvB4070AeGFP, respectively. The subsequent letter or number denotes mutants emerging in NIH-3T3 (N), HEK293 (2), U87-MG (U) or solid tumours (T), respectively. The final digit indicates the order of appearance of the PCR-fragment. In the case of revertants composed of more than one deletion, numbers and letters describing the participating recombinations are shown. Vector mutants which appear to have arisen consecutively contain the name of the putative precursor vector mutant in parentheses. White arrows indicate the size of the PCR product resulting from amplification of the respective parental vector. N = DNA from uninfected cultured cells or tumour tissue. Empty lanes indicate either that DNA isolation was unsuccessful or that DNA was isolated from tumour tissue apparently not infected. M = molecular size marker. Agarose gel images from PCR performed on *in vivo *infected tumour material 20d post-infection amended from Journal of Virology, July 2007, p. 6973–6983, Vol. 81, No. 13, doi:10.1128/JVI.02470-06, with permission from American Society for Microbiology.

The real-time RT-PCR and real-time PCR assays allow the exact quantification of the proportion of packaged vector RNA or proviral DNA lacking transgene sequence elements obligatory for PCR amplification, but do not permit the precise localisation and characterisation of individual recombination events leading to the generation of such mutants. Thus, alterations in the transgene cassette of vector mutants were detected by PCR of genomic DNA extracted from infected cells at the end of each infection cycle (Fig. 3A) and from infected tumours 5, 10, and 20 days following vector inoculation, as previously described [35] (Fig. 3B), using primers annealing to sequences in the *env *gene and in the 3' LTR. With this method, vector mutants which replicate more rapidly than the parental vector, and which have thus accumulated in the viral population, can be visualised as distinct bands.

In cells infected with ACE-GFP, bands representing vector recombinants were detectable following 17, 7, and 19 infection cycles of propagation in HEK293, NIH-3T3, and U87-MG cells, respectively (Fig. 3A), whereby the bands detectable in HEK-293 and U87-MG cells were much fainter than the bands representing parental vectors, indicating that deletion mutants only made up a small proportion of the replicating vector population. Bands representing vector mutants were detectable in all cell lines infected with vector ACEU3-GFP at a much earlier time point, on the other hand, following 13, 4, and 3 infection cycles in HEK293, NIH-3T3 and U87-MG cells, respectively (Fig. 3A), and, in each case, bands representing parental vectors became undetectable, indicating that mutant vectors had become dominant in the replicating vector population. Bands representing vector recombinants could be detected following 15, 9, and 11 infection cycles of vector AkvB4070AeGFP in HEK293 and U87-MG cells, and of vector Akv4070AeGFP in NIH-3T3 cells, respectively (Fig. 3A), with a concomitant decrease in the intensity of bands representing parental vectors also indicating that mutant vectors had become dominant in the replicating vector population. The accumulation of vector mutants detectable by PCR in this assay reflects the decrease in the ability of vectors to propagate a functional transgene cassette, as was previously shown by FACS analysis [35], indicating that loss of eGFP-expression is primarily due to recombination-mediated deletion and not to point mutations. Taken together, the differences in the appearance of bands representing mutant vectors indicate that (i) vector recombinants predominating in the replicating virus population emerge most rapidly in cells infected with vector ACEU3-GFP, (ii) this process occurs most slowly in cells infected with vector ACE-GFP, and (iii) the rate of emergence of predominant vector mutants was slowest, regardless of vector design or virus strain, when propagated in HEK293 cells. In *in vivo *infected tumours, bands representing vector mutants were infrequently detected except in tumours excised 20 days following infection with vector ACEU3-GFP (Fig. 3B), and, even here, bands representing parental vectors were also of similar intensity, indicating that deletion mutants had not become dominant in the vector population. As in infected cell cultures, it is apparent that the rate of emergence of predominant vector mutants was slowest in tumours infected with vector ACE-GFP and most rapid in tumours infected with vector ACEU3-GFP. Although several distinct bands could be detected corresponding to vector mutants which contained deletions of different sizes, no discernable pattern or trend regarding the size of the deletion mutants was revealed, and hence there seems to be no influence of the virus strain, transgene position, or host cell in this respect.

### Molecular mechanism of the emergence of vector recombinants

**Figure 4:**
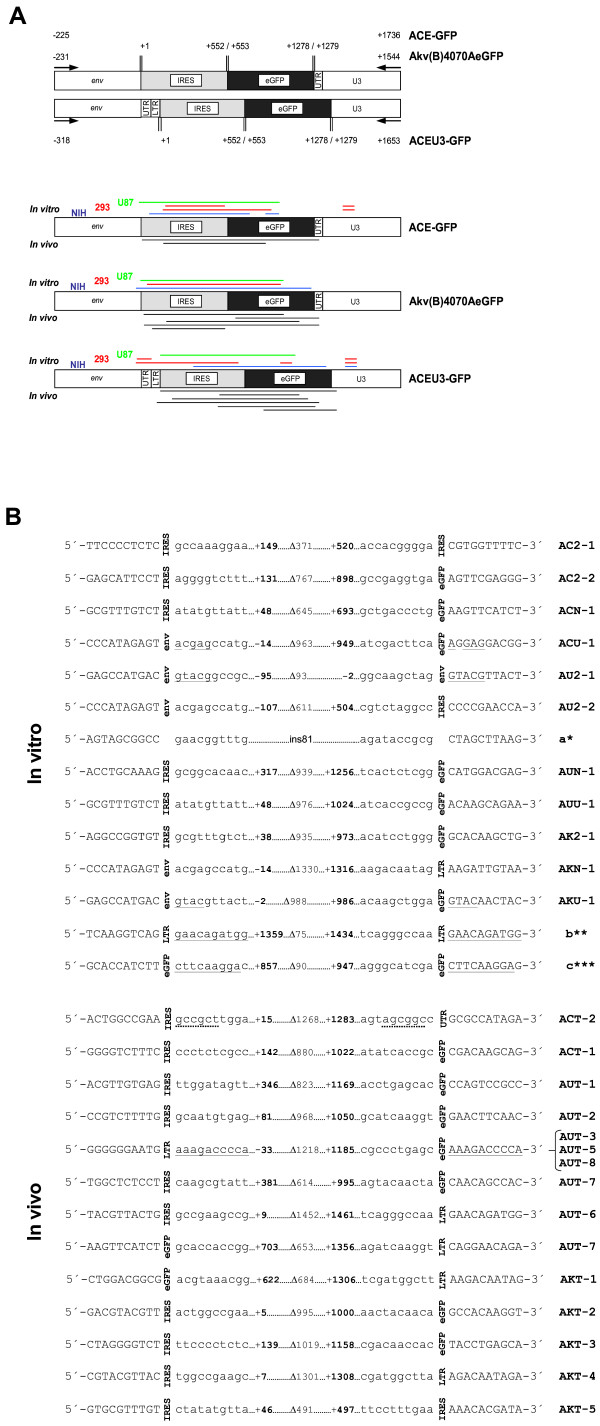
**Distribution and sequence analysis of vector recombinants**. **(A) **Schematic representation of vector mutants detected by PCR using primers annealing in the MLV 4070A *env *and *eGFP *gene indicating the distribution of the deleted sequences. Numbers indicate distances of vector border regions from the 5' end of the IRES. Annealing positions of sequencing primers are indicated by black arrows. Coloured horizontal lines indicate the positions of single deleted sequences in mutants isolated from *in vitro *infected cell lines (NIH-3T3 (blue), HEK293 (red), U87-MG (green)) and from *in vivo *infected U87-MG tumours (black). **(B) **Sequence analysis of deletion junctions. Nomenclature of the PCR products is according to Fig. 3. Intact (capital letters) and deleted sequences (lower case letters) are separated by vertical text indicating the regions where the deletions occurred. Bold numbers display the distances of the 5' and 3' deletion junctions from the 5' end of the IRES, which is set as 1. Lengths (nt) of deleted regions are indicated by a delta symbol. Repeats flanking the deleted sequences are indicated by underlined letters (continuous lines represent direct repeats, dotted lines represent inverted repeats). a* = insertion of 81 nt representing the 5' part of the 3' LTR U3 region, translocated from the 5' end of the transgene cassette and reconstituting the U3 region, b** = recurring 75 nt deletion occurring in the U3 region, detected in recombinants AC2-1, AC2-2, AU2-1, AU2-3, AU2-4, and AUN-1, c*** = 90 nt deletion found in mutant vectors ACN-2 and AU2-4.

To evaluate the role of previously described molecular determinants of retroviral recombination such as the prevalence of nucleotide sequence similarity [36,37] and RNA secondary structure [38-40,67] on the generation of vector recombinants which emerged in the replicating virus population, a total of 29 PCR products, representing the majority of vector recombinants which were detectable by PCR analyses, were excised from agarose gels and sequenced. Vector recombinants were classified with respect to the parental vector, the host cell line or tumour in which they emerged, the length and distribution of the characterised deletions, and the presence of motifs known to influence retroviral recombination rates (Fig. 4A and 4B). In total, 31 recombination junctions were sequenced, 16 and 15 of which were derived from vector recombinants which emerged during *in vitro *and *in vivo *experiments, respectively (Fig. 4B, Table 1). Interestingly, several deletions leading to the emergence of vector recombinants were detected repeatedly, not only in both Mo-MLV-based vectors ACE-GFP and ACEU3-GFP, but also in different cell lines, both *in vitro *and *in vivo*. However, no common deletion junctions were revealed when the recombination junctions of vector mutants emerging in *in vitro *and *in vivo *experiments were compared (Fig. 4B).

**Table 1:** Distribution of 5' recombination junctions in vector mutants isolated *in vitro *and *in vivo *with respect to flanking nucleotide sequence homology and EMCV IRES secondary structure

	**in vitro**^a^				**in vivo**^b^	**TOTAL**
	
	NIH-3T3	HEK293	U87-MG	TOTAL		
			
**within IRES**	2	3	1	6	10	16
paired^c^	1	0	0	1	7	8
unpaired^d^	1	3	1	5	3	8
≥ 4 nt repeat^e^	0	0	0	0	0	0
**outside IRES**	3	5	2	10	5	15
≥ 4 nt repeat	2	4	1	7	3	10

**TOTAL**	5	8	3	16	15	31

^a^Distribution of 5' recombination junctions in vector mutants isolated from *in vitro *infected NIH-3T3, HEK293, and U87-MG cells at the end of each cycle

^b^Distribution of 5' recombination junctions in vector mutants isolated from pre-formed subcutaneous U87-MG tumours growing in nude mice and infected *in vivo*

^c^5' recombination junctions which fall within paired regions of the EMCV IRES RNA secondary structure [44,67]

^d^5' recombination junctions which fall within unpaired regions of the EMCV IRES RNA secondary structure [44,67]

^e^5' recombination junctions which are flanked by nucleotide sequence homology (direct or inverted repeats) of = 4 nt in length

In *in vitro *infections, both NIH-3T3 cells transduced with vector ACE-GFP and HEK293 cells infected with vector ACEU3-GFP gave rise to vector mutants containing a 90 nucleotide (nt) deletion flanked by a 18 out of 19 nt imperfect direct repeat in the middle of the *eGFP *gene (deletion c, occurring in mutants ACN-2 and AU2-4, respectively, Fig. 3A and 4B). Another recombination event which was repeatedly detected in mutants which emerged in cells infected with the Mo-MLV based vectors led to the deletion of 75 nts of the U3 region, corresponding to one of the two tandem copies of a transcriptional enhancer element [41] (deletion b, occurring in mutants AC2-1+b, AC2-2+b, AU2-1+a+b, AU2-2, AU2-4 and AUN-1+b, Fig. 3A and 4B), presumably mediated by a 10 nt perfect direct repeat flanking the deletion junctions. Interestingly, several of the mutants which were characterised also revealed a translocation of 81 nts including the T-stretch [42], the primer binding site and the first 31 nts of the U3 region, leading to the reconstitution of wild type Mo-MLV LTR sequences (insertion a, occuring in mutants AU2-1+a+b, AU2-2+a, AU2-3 and AU2-4, Fig. 3A and 4B). Furthermore, mutants which contained deletions associated with recombination events originating outside of transgene sequences were not confined to non-translated regions of the MLV genome. In both Mo-MLV-based vectors and also in the N-tropic Akv-MLV-based vector, recombinants emerged which shared the same 5' deletion junction, whereby the 3' terminal 11 nts (including the stop codon) of the *env *gene were deleted (mutants ACU-1, AU2-2, and AKN-1, Fig. 3A and 4B).

In several infected cell lines, the emergence of vector mutants could be detected which appeared to be the product of consecutive recombination events. In NIH-3T3 cells infected with vector ACE-GFP, for example, a recombinant (mutant ACN-1) emerged at infection cycle 7, and seems to have undergone further recombination to generate a second mutant, ACN-2, which emerged at infection cycle 13 (Fig. 3A). Similar events could be observed for vectors ACE-GFP and ACEU3-GFP in HEK293 cells (Fig. 3A).

To provide an overview of the sequence motifs involved in recombination leading to deletion of transgene sequences facilitating the emergence of vector mutants, each characterised mutant was classified with respect to position of the 5' recombination junction, and to the presence of nucleotide homology and/or RNA secondary structure [44,67], in sequences flanking the recombination junctions (Table 1). The majority of all characterised deletion mutants containing the 5' deletion junction in non-IRES sequences were flanked by direct repeats, whereas all of the mutants containing the 5' junction within the IRES sequence were mediated by non-homologous recombination (Table 1). No differences concerning the types of recombination events leading to the emergence of vector mutants between vectors of different strain or transgene insert position could be discerned, however.

Of the sequenced deletion junctions derived from mutants which emerged during *in vitro *experiments, 6 (37.5%) involved 5' recombination sequences within the EMCV IRES RNA and 10 (62.5%) used 5' recombination sequences in non-IRES sequences, of which 7 were flanked by perfect or imperfect direct repeats of 4 nts or longer (Table 1). Of the 6 deletion junctions which used 5' recombination sequences within the IRES, 5 had 5' junctions positioned within an unpaired region of the RNA secondary structure [43,44,67] and were formed predominantly via non-homologous recombination (Table 1). Of the 15 junctions derived from vector mutants which emerged in infected tumours, 10 utilised 5' recombination sequences within the EMCV IRES and 5 used 5' recombination sequences in non-IRES sequences, of which 3 involved identical perfect direct repeats of 10 nts (mutants AUT-3, AUT-5 and AUT-8, Fig. 3B and 4B, Table 1). Interestingly, of the 10 vector mutants which emerged *in vivo *containing 5' recombination junctions positioned in the IRES, only 3 contained the 5' recombination junction within an unpaired region of the IRES RNA secondary structure, suggesting that the precise mechanisms leading to the emergence of vector mutants in cell culture and in solid tumours may differ.

## Discussion

A number of MLV-based RCR vector designs which hold great promise for use in cancer gene therapy applications have recently been described, demonstrating a therapeutic efficacy in experimental tumours far exceeding that of equivalent replication-deficient vectors (for review see [45]). High genomic stability, however, is of central importance for the future clinical success of RCR vectors, since only dissemination of the intact transgene cassette to the entire tumour mass can guarantee that each malignant cell will be exposed to the therapeutic effect.

Thus, in the present study, we analysed the rate of emergence of vector recombinants in cells and solid tumours infected with a panel of MLV-based RCR vectors of different designs, and characterised the molecular mechanisms involved in the associated recombination processes. In general, the rate of emergence of predominating vector recombinants correlated with the reduction in the percentage of eGFP-positive cells during iterative propagation cycles of the respective vectors on cultured cells (Fig. 2A) [35].

The greatly superior genomic stability of vectors containing an IRES-eGFP cassette fused to the 3' end of the *env *gene compared to when inserted into the U3 region of the 3' LTR is likely due to insertion of the transgene in the U3 region impairing virus replication to a greater extent.

Reasons for the observed differences in the genomic stability of vectors based on different MLV strains and in different cell types are more ambiguous. Seemingly the most obvious reason for decreased genomic stability of an MLV-based RCR vector in NIH-3T3 cells would be recombination with an endogenous retrovirus, as has been previously reported in studies of MLV-based RCR vectors containing ecotropic *env *genes [46]. However, sequencing of the recombination junctions of vector mutants did not reveal any role for this in the present study, perhaps because the amphotropic *env *gene was used. Amphotropic envelope sequences are not present in the Mus germ line [47] and hence, presumably, vectors containing the amphotropic *env *gene are less likely to recombine via homologous recombination with endogenous *env *sequences than vectors containing ecotropic *env *genes. On the other hand, it is important to bear in mind that only recombinants which maintain binding sites for the PCR primers will be detected, hence, recombinants which may contribute to the overall instability of the replicating viral vector population may have been overlooked by the PCR-based assay used in this study. In addition, if recombination events did indeed occur with endogenous env sequences, it is possible that the resulting recombinant vectors would also remain undetected within the replicating viral population. This would also, presumably, be more likely to occur with Akv-MLV based vectors, since Akv-MLV is itself a member of the closely related ecotropic MLV family.

At first glance, it would also appear that the genomic stability of vectors decreases as a function of their replication kinetics in a particular cell line, since, as previously described, all vectors replicate most rapidly in NIH-3T3 cells, in which they are also the least stable [35]. However, this would not explain the differences, for example, in the stability of vectors ACEU3-GFP or Akv(B)4070AeGFP in U87-MG and HEK-293 cells, in which their replication kinetics are more similar to each other. Moreover, we have also performed studies in human HepG2 cells [24], where we also found vector ACE-GFP to be much less stable when compared to the results obtained here in U87-MG and HEK-293 cells, in spite of the fact that ACE-GFP replication kinetics in HepG2 cells are similar to, or slower, than in HEK-293 cells. Hence, the reasons for the differences in vector genomic stability between cell lines seems to be neither species specific nor directly related to the different replication kinetics of the vectors in the respective cell lines.

The observed differences in vector stability in different cell lines could, on the other hand, potentially be due to differences in other virus and/or host-cell features. The intrinsic fidelity of the RT of the Akv and Moloney strains of MLV for example, and/or their nucleocapsid (NC) proteins may differ in the efficiency of modulating the reverse transcription reaction, as has been demonstrated for the RT from different types of retrovirus [48-51]. Cellular RNAP II, which synthesizes the retroviral genome, has also been demonstrated to introduce mutations and/or deletions into retroviral vector genomes, albeit at a low frequency [7], and it is possible that some cell line specific differences in the fidelity of this process exist. Additionally, p53 expression has been shown to be able to provide a proof-reading function for reverse transcriptase [52-54], and it is possible that this could also play a role in the differences in genomic stability of vectors in different cell lines, as could also be hypothesized for differences in the expression of host-encoded anti-viral mechanisms such as APOBEC and TRIM family members [55-58]. Host cell-specific differences in vector genomic stability could also be influenced by the availability and balance of intracellular dNTP pools, as described for cells with low metabolic activity or under hypoxic conditions, since it is known that this can decrease the processivity of RT and concomitantly increase the frequency of RT-pausing and recombination [59-61], as can low temperatures [62,63].

Vector genome motifs which have been demonstrated to increase the frequency of recombination events include nucleotide sequence homology (direct and indirect repeats) [36,37], homopolymeric sequences [64-66], and RNA secondary structure [38-40]. In the present study, PCR and sequencing of vector mutants emerging in infected cells and tumours revealed that the majority of vector deletions in which the 5' deletion junction was positioned in non-IRES sequences were flanked by direct repeats (Fig. 4B, Table 1). These results underpin those of Logg and colleagues, who found that all sequenced vector mutants emerging in Mo-MLV-based RCR vectors containing transgene cassettes in the 3' UTR were associated with flanking nucleotide homology [46]. Moreover, our results revealed no virus-strain specific transgene deletion mechanism bias, which is important, since the only other study on recombination events in RCR vectors utilised the Akv strain of MLV [67]. In this study, the authors detected an RNA secondary structure bias for recombination events leading to the emergence of vector mutants, albeit only deletion mutants with 5' recombination junctions in the EMCV IRES were taken into account. In the present study, we also found that the majority of 5' recombination junctions positioned in the EMCV IRES in vector mutants emerging in cell culture were located in unpaired regions of RNA, underpinning the results of Duch and colleagues, and demonstrating that this observation is not restricted to the Akv strain of MLV. Interestingly, however, only 30% of the 5' recombination junctions within the IRES sequence were positioned in unpaired RNA secondary structures in the analysed vector mutants emerging *in vivo*, in contrast to over 75% in cell culture, when our studies and those of others [46,68] are combined. This result suggests that there may be differences in the mechanisms leading to the emergence of recombinant vectors in cell culture and solid tumours. However, although we sequenced and analysed the vast majority of deletion mutants which we could detect by PCR in this study, amounting to a total of 15 vector mutants detected in 45 xenograft tumours, this conclusion remains tentative due to the relatively small number of recombination events which we could analyse. It remains to be seen whether our observations will be supported by data arising from future analyses of RCR vectors in solid tumours.

Common recombinant junctions were invariably associated with flanking nucleotide sequence homology, either within the *eGFP *gene, or within the U3 region of the Mo-MLV LTR, whereby the latter was involved in the generation of mutants lacking one copy of a tandem enhancer sequence [41]. While it is not expected that the deletion of viral sequences should generate mutants of increased replicative fitness, spontaneous enhancer deletions have previously been described [69], and it may be that a second copy of the enhancer sequence is redundant for efficient replication in certain cell types.

Interestingly, in both Mo-MLV- and Akv-MLV-based vectors, deletion mutants emerged in which short sequences at the 3' end of the *env *gene were deleted, resulting either in truncated or elongated Env proteins. It has previously been demonstrated that short truncations of the C-terminus of the Env protein have no impact on cell-cell fusion or viral transduction [70] and that the actual composition of the R-peptide, located at the very C-terminal end of the Env protein, has minimal effect on the inhibition of its fusogenic potential [71]. Hence it can be assumed that the relatively short truncations and elongations of the Env protein resulting from the deletions observed in this study also had no detrimental effect on the replicative fitness of the affected mutants. A further unusual recombination event, occurring in vector ACEU3-GFP, involved the translocation of 81 nts of the 3' UTR and U3 region, and led to the reconstitution of a wild type Mo-MLV LTR, indicating that the reduced genomic stability of ACEU3-GFP compared to ACE-GFP may be a result of the interruption of the spatial integrity of the 3' UTR and U3 region by the IRES-eGFP cassette. Such translocation events leading to restoration of wild-type retroviral LTRs have been previously described for U3-deleted SNV vectors, where it was shown that vectors with a deleted 3' LTR U3 region underwent recombination between the 5' and 3' LTRs to restore wild-type U3 sequences in both LTRs [72].

## Conclusion

Taken together, our data provide additional evidence that the high selection pressure imposed on MLV-based RCR vectors to delete transgenic sequences which interfere with their replication kinetics can be overcome to an extent by using appropriate vector designs. Clearly, results obtained using a non-therapeutic marker gene such as eGFP in cell culture or in tumour models in nude mice do not truly reflect the situation which will be encountered in a therapeutic situation in immunocompetent patients. The potential effects of therapeutic transgene toxicity and immunogenicity, as well as additional unpredictable influences of specific transgene sequences on RCR replication kinetics and genomic stability, must therefore be determined on a case-by-case basis in cell culture and animal models before embarking on a clinical study. This notwithstanding, the PCR, sequencing and real time PCR/RT-PCR analyses in the present study strongly support the hypotheses suggested by our analyses of functional transgene propagation [35], that MLV-based RCR vectors based on the Moloney strain of MLV and containing the transgene cassette in the 3' UTR region are more suitable for potential cancer gene therapy applications than those based on Akv-MLV and/or containing the transgene cassette in the U3 region of the LTR. Our findings that, in cell culture, deletion mutants which emerge are generally associated with nucleotide homology or unpaired regions of RNA secondary structure reinforces similar observations obtained from more delimited studies of recombination mechanisms associated with MLV-based RCR vectors [46,68], indicating that vector stability could possibly be further improved by reducing the frequency of such motifs in the transgene cassette. Moreover, to our knowledge, this is the first study which has investigated the underlying mechanisms associated with transgene deletion in RCR vector recombinant emergence *in vivo*, and the discrepancies between the data obtained in solid tumours *in vivo *and our own and previously published data from infected cells *in vitro *demonstrates the importance of evaluating vectors designed for use in cancer gene therapy *in vivo *as well as *in vitro*.

## Methods

### Plasmids, cell culture and virus production

Plasmids pACE-GFP [22], pACEU3-GFP, pAkv4070AeGFP and pAkvB4070AeGFP [35], encoding replication-competent retroviral vectors of different strain and transgene insertion site were used for transient transfection of HEK293 cells (ATCC CRL-1573) to produce viral vector stocks as described previously [35]. The B-tropic vector pAkvB4070AeGFP differs from N-tropic vector pAkv4070AeGFP only by a Glu to Arg transition in the capsid protein, permitting efficient infection of human cells.

Subsequent infection cycles on HEK293 cells, NIH-3T3 cells (ATCC CRL-1658) and U87-MG cells (ATCC HTB-14) were performed as previously described [35]. Briefly, cells were transduced with the respective vectors at an multiplicity of infection (MOI) of 0.001 to initiate infection cycle 1, two days later the infected cells were passaged 1:3, and an aliquot was used to measure enhanced green fluorescence protein (eGFP) expression via fluorescence activated cell sorting (FACS). Four days post infection serial dilutions of supernatant from infected cells were used to initiate infection cycle 2. Cells infected with a dilution allowing exponential vector spread up to 4 days post infection were used to continue the experiment. Infected cells were passaged every 2 or 3 days until the percentage of eGFP-positive cells ceased to increase from one passage to the next. Serial infection cycles were continued in this manner until less than 2% of infected cells became eGFP-positive following serial passaging. At the end of each infection cycle, cell-free virus-containing supernatants and cell pellets were stored by freezing at -80°C.

For *in vivo *infection, preformed U87-MG subcutaneous tumours growing in 4-week-old male Hsd:athymic Nude-*Foxn1*^*nu *^mice (Harlan) were used, as described previously [35]. All animal experiments were discussed and approved by the institutional ethics committee (GZ 68.205/53-BrGt/2004). Briefly, at a tumour size of 60 to 100 mm^3^, normally three to four weeks after cell injection, 1 × 10^5 ^viral particles suspended in 20 μl Dulbecco's modified Eagles medium (DMEM, Invitrogen) were injected directly into the tumour centre. Five mice per vector and time point were sacrificed 5, 10, and 20 days post infection, and tumours were excised and used for further analysis.

### Real-time PCR, real-time RT-PCR, and PCR analysis

Genomic DNA and viral RNA was extracted from infected cells and tumour tissue, and from virus-containing supernatants, respectively, using the DNeasy Tissue Kit (Qiagen) or the Viral RNA Kit (Qiagen) as recommended by the manufacturer. PCR amplification of vector sequences was performed using primers annealing to sequences in the amphotropic MLV 4070A *env *gene and in the 3'-LTR [35]. Real-time PCR and real-time RT-PCR was performed using primers and probes annealing either to sequences in the 4070A *env *gene or in the encephalomyocarditis virus (EMCV) internal ribosome entry site (IRES) or the *eGFP *gene. Primers and probes were designed with Primer Express 3.0 software (PE Applied Biosystems) and synthesized by Microsynth. Primers and probes annealing to *eGFP *and *env *gene sequences have been described previously [35,73]. For detection of IRES-specific sequences, primers IRES-F (5'-AACAACGTCTGTAGCGACCCTT-3') and IRES-R (5-GCCGCCTTTGCAGGTGTAT-3'), and probe IRES-P (5'-CTGGCGACAGGTGCCTC TGCG-3') were used. Real-time PCR and real-time RT-PCR reactions were performed as described elsewhere [73].

### Sequence analyses

PCR products were separated by agarose gel electrophoresis, isolated and purified from the agarose gel using the QIAquick Gel Extraction Kit (Qiagen) according to the manufacturer's protocol, and either sequenced directly using primers employed for genomic DNA amplification, as described above, or subcloned into vector pCR2.1 (Invitrogen) and sequenced using standard primers M13 forward (5'-TTCCCAGTCACGACGTTG-3') and M13 reverse (5'-CACACAGGAAACAGCTATGACC-3').

## Abbreviations

MLV: murine leukaemia virus; RCR: replication competent retroviral; RT: reverse transcriptase; RDR: replication deficient retroviral; eGFP: enhanced green fluorescent protein; FACS: fluorescence activated cell sorting; DMEM: Dulbecco's Modified Eagle Medium; RT-PCR: reverse transcriptase-polymerase chain reaction; EMCV: encephalomyocarditis virus; IRES: internal ribosome entry site; env: envelope; Mo: Moloney; UTR: untranslated region; LTR: long terminal repeat; nt: nucleotide; U3: unique 3' region; U5: unique 5' region; R: repeat region; dNTP: deoxyribonucleoside triphosphate.

## Authors' contributions

MP was responsible for performing cell culture and *in vivo *experiments and drafting the manuscript. DK assisted with real time PCR and real time RT-PCR assay establishment and data analysis. BS and WHG contributed to conception of the study design. MR assisted in conception and supervision of the study design and drafting the manuscript. DP conceived and supervised the study design and drafted the manuscript. All authors read and approved the final manuscript.
